# Impact of heart failure on the behavior of human neonatal stem cells *in vitro*

**DOI:** 10.1186/1479-5876-11-236

**Published:** 2013-09-27

**Authors:** Kristin Klose, Rajika Roy, Andreja Brodarac, Andreas Kurtz, Andrea Ode, Kyung-Sun Kang, Karen Bieback, Yeong-Hoon Choi, Christof Stamm

**Affiliations:** 1 Berlin-Brandenburg Center for Regenerative Therapies, Charité-University Medicine Berlin, Augustenburger Platz 1, 13353 Berlin, Germany; 2 Julius Wolff Institute and Center for Musculoskeletal Surgery, Charité-University Medicine Berlin, Augustenburger Platz 1, 13353 Berlin, Germany; 3 Adult Stem Cell Research Center, College of Veterinary Medicine, Seoul National University, 599 Gwanakno, Sillim-Dong, Seoul 151-742, South Korea; 4 Medical Faculty Mannheim, Institute of Transfusion Medicine and Immunology, Heidelberg University, Friedrich-Ebert-Str. 107, 68167 Mannheim, Germany; 5 Heart Center, Cardiothoracic Surgery and Center for Molecular Medicine Cologne, University of Cologne, Kerpener Str. 62, 50924 Köln, Germany; 6 Deutsches Herzzentrum Berlin, Augustenburger Platz 1, 13353 Berlin, Germany

**Keywords:** Heart failure, Human, Mesenchymal stromal cells, Cord blood, Proliferation, Serum

## Abstract

**Background:**

Clinical cardiac cell therapy using autologous somatic stem cells is restricted by age and disease-associated impairment of stem cell function. Juvenile cells possibly represent a more potent alternative, but the impact of patient-related variables on such cell products is unknown. We therefore evaluated the behavior of neonatal cord blood mesenchymal stem cells (CB-MSC) in the presence of serum from patients with advanced heart failure (HF).

**Methods:**

Human serum was obtained from patients with severe HF (n = 21) and from healthy volunteers (n = 12). To confirm the systemic quality of HF in the sera, TNF-α and IL-6 were quantified. CB-MSC from healthy neonates were cultivated for up to 14 days in medium supplemented with 10% protein-normalized human HF or control serum or fetal calf serum (FCS).

**Results:**

All HF sera contained increased cytokine concentrations (IL-6, TNF-α). When exposed to HF serum, CB-MSC maintained basic MSC properties as confirmed by immunophenotyping and differentiation assays, but clonogenic cells were reduced in number and gave rise to substantially smaller colonies in the CFU-F assay. Cell cycle analysis pointed towards G1 arrest. CB-MSC metabolic activity and proliferation were significantly impaired for up to 3 days as measured by MTS turnover, BrdU incorporation and DAPI + nuclei counting. On day 5, however, CB-MSC growth kinetics approached control serum levels, though protein expression of cell cycle inhibitors (p21, p27), and apoptosis marker Caspase 3 remained elevated. Signal transduction included the stress and cytokine-induced JNK and ERK1/2 MAP kinase pathways.

**Conclusions:**

Heart failure temporarily inhibits clonality and proliferation of “healthy” juvenile MSC *in vitro*. Further studies should address the *in vivo* and clinical relevance of this finding.

## Background

Cardiac cell therapy is currently being evaluated as a treatment option for patients with severe heart failure (HF) (reviewed in [1]). In contrast to experimental models of heart disease, cell-based therapy in humans has brought about only moderate improvements in heart function and there is very little evidence of *de novo* formation of cardiomyocytes. This mismatch between animal experiments and human clinical trials may be explained by age and disease-related impairment of stem cell function in HF patients [2,3]. To circumvent the limited regenerative capacity of autologous stem cells from elderly and chronically sick patients, neonatal cell products from healthy donors have been suggested as a superior alternative. However, it has not yet been investigated whether these juvenile cells would withstand the harsh environment upon transplantation into a failing heart. Apart from ischemia and pathologic extracellular matrix architecture, HF may affect stem cell function through circulating disease-related humoral factors. An abnormal molecular milieu is present in HF [4], and several publications have pointed to a role for HF-associated circulating factors in the modulation of adult stem cell function: Yamahara *et al.,* for instance, identified Angiostatin as an inhibitor of human bone-marrow-derived mesenchymal stem cell (hBM-MSC) proliferation and migration; and Gatta *et al.* showed that serum composition determines growth of colony-forming endothelial progenitor cells (CFU-EC) [5,6]. The influence of humoral factors on neonatal stem cell function, however, is still unknown. We therefore sought to test the hypothesis that, *in vitro,* HF serum factors impair the functionality of neonatal cord blood mesenchymal stem cells (CB-MSC). We chose neonatal CB-MSC from healthy donors because this cell type is characterized by young chronological age and absent disease-associated functional impairment, and possesses a marked proliferation capacity and a broad differentiation potential (reviewed in [7]). In summary, we found that heart disease does have an impact on CB-MSC biology as evident from impaired proliferation characteristics, stimulation of apoptosis and activation of stress signaling pathways.

## Material and methods

### Study population: heart failure patients and healthy control subjects

The study was done in accordance with the Declaration of Helsinki, with approval of the ethics committee of Charité-University Medicine Berlin, and with informed consent of all patients and volunteers. Blood samples were collected from patients with chronic HF (n = 21) during hospitalization. Patients were included in the study if they had left ventricular ejection fraction (LVEF) <40% as determined by echocardiography, with New York Heart Association (NYHA) functional class III or IV, and an indication for cardiac surgery including implantation of a left ventricular assist device. Patients were excluded if they were not clinically stable or had cancer, any active infection or an indication for heart transplantation. The clinical details are summarized in Table 1. Blood samples were also taken from 12 healthy control subjects. Detailed information on those is also provided in Table 1.

**Table 1:** Characteristics of healthy controls and HF patients

	**Healthy control subjects (n = 12)**	**Heart failure patients (n = 21)**
**Demographic data and medical history**		
Age [years]	40 (3, 24–71)	60 (12, 31–82)
Sex (male/female)	6/6 (50%/50%)	15/6 (71%/29%)
Smoking status	4 (25%)	9 (43%)
HF duration [month]	-	13 (4.6, 0.3-48)
Previous surgery	-	0
Previous MI	-	13 (62%)
Time since most recent MI [month]	-	2.7 (1.8, 0.1-36)
Extent of CAD		
One vessel	-	0
Two vessels	-	0
Three vessels	-	21 (100%)
NYHA functional class		
III	-	13 (62%)
IV	-	8 (38%)
Heart rate [bpm]	-	92 (13)
Systolic blood pressure [mmHg]	-	118 (26, 82–167)
Diastolic blood pressure [mmHg]	-	76 (19, 54–104)
**Etiology**		
Ischemic	-	16 (76%)
Non-ischemic	-	5 (24%)
Idiopathic	-	0
**Echocardiographic data**		
LVEDD [mm]	-	153 (38, 93–232)
LVESD [mm]	-	73 (26, 58–149)
LVEF [%]	-	24.7 (13.4, 15–38)
**Surgery**		
Ventricular assist device	-	5 (24%)
Bypass graft	-	12 (57%)
Valve	-	5 (24%)
**Non-cardiac disease**		
Allergy	1 (8%)	4 (19%)
S.p. apoplexia	1 (8%)	1 (5%)
Hypertension	3 (25%)	17 (81%)
Osteoporosis	1 (8%)	3 (14%)
Hypothyroidism	2 (17%)	0
Diabetes type II	1 (8%)	11 (52%)
Chronic obstructive pulmonary disease	1 (8%)	3 (14%)
**Pharmacological therapy**		
Aspirin	1 (8%)	12 (57%)
Heparin	0	15 (71%)
Coumadin	1 (8%)	3 (14%)
Statins	1 (8%)	14 (67%)
Loop diuretics	2 (17%)	15 (71%)
β-blockers	1 (8%)	5 (24%)
Digitoxin	0	8 (38%)
Metformin	1 (8%)	0
ACE Inhibitors	1 (8%)	17 (81%)
Amniodarone	0	6 (29%)
Amlodipine	1 (8%)	0
Biphosphonates	1 (8%)	0
Pantoprazole	0	18 (86%)
Catecholamines	0	6 (29%)

Data is expressed as number (percent) or as mean (SD, range).

ABBREVIATIONS: *MI* myocardial infarction, *CAD* coronary artery disease, *NYHA* New York Heart Association, *LVEDD* left ventricular end-diastolic diameter, *LVESD* left ventricular end-systolic diameter, *LVEF* left ventricular ejection fraction.

### Human serum preparation

Blood was drawn by venipuncture into S-Monovettes® (Sarstedt, Nümbrecht, Germany) using the BD Vacutainer Safety-Lok™ blood collection set (BD Medical, Heidelberg, Germany). Serum off the clot was obtained from whole blood which underwent the natural clotting process. Since HF patients routinely receive anticoagulants, blood samples were subjected to a prolonged clotting period (3 h as compared to 30 min: 20 min at room temperature (RT) to initiate the clotting process followed by 2 h 40 min on ice to prevent degradation of serum constituents). After centrifugation for 15 min at 3500 g and 4°C, serum supernatants were sterile filtered. Aliquots were flash frozen and stored in liquid nitrogen, because it took several weeks to recruit enough patients so that experiments with multiple sera could begin. Hemolytic tubes were excluded.

### Protein normalization of human serum

The protein content of human blood varies with a number of parameters at the time of blood sampling, such as liquid intake, physical activities, drugs, disease status, and previous infusions [8]. To account for this, serum protein concentration was determined by Bradford assay (Carl Roth, Karlsruhe, Germany) including interpolation from a standard curve established with bovine serum albumin (BSA), and normalized to the lowest concentration (~37 mg/ml, which corresponds to ~10% FCS supplementation) by respective dilution in Gibco® D-PBS with Ca^2+^ and Mg^2+^ (PBS^++^) (Life Technologies, Darmstadt, Germany).

### Cytokine ELISA

To make sure that sera were indeed typical for patients with chronic HF, selected cytokines were determined using high sensitivity colorimetric assays: Human IL-6 Quantikine HS ELISA Kit (RnD Systems, Wiesbaden-Nordenstadt, Germany) and Human TNF-α Quantikine HS ELISA (RnD Systems). All immunoassays were performed according to the manufacturer’s instructions. Human serum samples were thawed and protein normalized immediately before the assay.

### Standard cell culture of human CB-MSC

Cryopreserved human CB-MSC were provided by K.B., who isolated and expanded CB-MSC according to a previously published protocol [9]. Cord blood was obtained with informed consent of the mother, according to the principles outlined in the Declaration of Helsinki and with approval of the local ethical committees in Mannheim and Heidelberg (Ref. 48/05 reconfirmed in 2009). Passage (P) 2 cells were expanded in low glucose Gibco® DMEM (DMEM-LG) (Life Technologies) supplemented with 10% FCS (Life Technologies, pre-tested batch 41F6495K) and 1% penicillin/streptomycin (P/S) (Lonza, Basel, Switzerland). Cells were seeded at a density of ~1000 cells/cm^2^ in T175 flasks; partial medium change was given every 3 days. At subconfluency, cells were subcultured using Gibco® TrypLE™ Express (Life Technologies), or cryopreserved in FCS supplemented with 10% DMSO (Carl Roth) and stored in liquid nitrogen. Cells used for experiments originated from the umbilical cord of a single healthy donor and had undergone 2–3 freeze-thaw cycles and ~20 population doublings, referred to as P4 cells. Cell culture vessels employed throughout the study were either CELLSTAR® (Greiner Bio-One, Frickenhausen, Germany) or Nunclon™∆ Surface (Nunc, New York, NY, USA) products.

### *In vitro* system establishment and study design

Several criteria (CB-MSC initial seeding density, human serum concentration, exposure time, means and interval of serum supplementation, necessity for dual supplementation using FCS and human serum) were examined for the design of a suitable *in vitro* system. Unless otherwise stated, final conditions were as follows: Cryopreserved CB-MSC were thawed and plated at a density of 4000 cells/cm^2^ in medium supplemented with 10% FCS. After cell attachment, on day 0, FCS was replaced by 10% protein-normalized human serum. Complete medium change was performed every 2 days. Total exposure time was 5–14 days. Individual sera were not pooled, but tested separately. Human serum from healthy donors served as experimental control. In parallel, all experiments were also performed with cells maintained under standard culture conditions (FCS supplementation).

### MTS metabolic activity assay and BrdU proliferation assay

The CellTiter 96® Aq_ueous_ Non-Radioactive Cell Proliferation Assay (Promega, Mannheim, Germany) and the colorimetric BrdU Cell Proliferation ELISA (Roche, Mannheim, Germany) were performed according to the manufacturer’s instructions on days 1, 3 and 5 on triplicate wells of 96-well plates.

### DAPI-based direct cell counting

Subsequent to MTS assay, cells were fixed in 4% paraformaldehyde (PFA) (Carl Roth) and stained with Molecular Probes® DAPI (Life Technologies). The Operetta high content screener (PerkinElmer, Rodgau, Germany) was used to capture 23 10X-images per well that covered the whole culture surface. Harmony® software (PerkinElmer) was used for image analysis, which was performed to quantify CB-MSC numbers based on DAPI-stained CB-MSC nuclei. Since CB-MSC are single nucleated cells, nuclei counts served as direct cell counts to validate indirect proliferation readings acquired from colorimetric assays (MTS, BrdU).

### Phase contrast microscopy of cell morphology

Morphology of live unstained cells was examined by phase contrast microscopy on days 1, 3, 4 and 5 of human serum treatment.

### Flow cytometric immunophenotyping

Cell harvests were washed in PBS and blocked in FACS buffer (1% BSA, 0.05% sodium azide in PBS) for 15 min at 4°C. Triple combinations of antibodies were employed to stain 50,000 cells in 100 μl volume for 30 min at 4°C. Propidium iodide (PI) (BD Biosciences, Heidelberg, Germany) was added after 15 min to a final concentration of 5 μg/ml. Excess antibodies were washed off and cells were resuspended in FACS buffer and analyzed in a BD FACSCalibur flow cytometer (Becton Dickinson, NJ, USA). Fifteen thousand events were acquired and analyzed using FlowJo software (Tree Star, Ashland, OR, USA). The following mouse antibodies were used: Biozol (Eching, Germany): CD14-FITC, CD73-PE; Miltenyi Biotec (Bergisch Gladbach, Germany): CD34-PE, CD45-FITC; eBioscience (Frankfurt, Germany): CD90-APC; antibodies-online (Aachen, Germany): CD105-FITC, HLA-DR-FITC [10].

### Tripotential mesodermal differentiation

For osteogenic and adipogenic differentiation, CB-MSC were grown to subconfluency in the presence of 10% human HF or control serum or FCS in 6-well plates (for q-RT-PCR analysis) or 12-well plates (for stainings). After 7 days, osteogenic or adipogenic induction medium (StemPro® Osteocyte/Adipocyte Differentiation Basal Medium supplemented with StemPro® Osteogenesis/Adipogenesis Supplement, Life Technologies) was added and changed twice per week. After 21 days, cells were fixed and stained with Alizarin Red S or Oil Red O (Sigma-Aldrich, St. Louis, MO, USA) to confirm osteogenic (calcium deposits) or adipogenic differentiation (intracellular lipid granules). For chrondrogenic differentiation a 3-D culture system was used: After 7 days of treatment with 10% human HF or control serum or FCS, CB-MSC were subcultured. Approx. 3.5×10^5^ cells were pelleted in 15 ml tubes (for q-RT-PCR analysis), droplets containing 0.8×10^5^ cells were seeded into 12-well plates to generate micromass cultures (for stainings). Induction medium (StemPro® Chondrocyte Differentiation Basal Medium supplemented with StemPro® Chondrogenesis Supplement, Life Technologies) was added and changed twice per week. After 21 days, micromass cultures were fixed and stained with Alcian Blue (Carl Roth) to visualize proteoglycan deposits. Undifferentiated cells served as negative controls.

### Quantitative real-time PCR

Total RNA was extracted from undifferentiated (after 7 days of human serum treatment) and differentiated cells (after 7 days of human serum treatment and 21 days of differentiation) using the Qiagen RNeasy Mini Kit (Qiagen GmbH, Hilden, Germany). Contaminating genomic DNA was removed with DNAse I (Sigma-Aldrich). Subsequently, 100 ng of each RNA sample were random hexamer primed and reverse transcribed into cDNA using the Invitrogen™ SuperScript® III First-Strand Synthesis System (Life Technologies). Real-Time PCR was performed using the Applied Biosystems® Power SYBR® Green PCR Master Mix (Life Technologies) and the Eppendorf Mastercycler ep gradient S realplex^2^ (Eppendorf AG, Hamburg, Germany) by combining 12.5 μl SYBR Green master mix, 2 μl primer mix (forward:reverse 1:1, final concentration 800nM), 5.5 μl sterile ddH_2_O and 5 μl cDNA (1.25 ng template per reaction) per well in an Applied Biosystems® MicroAmp® Optical 96-Well Reaction Plate (Life Technologies). Each PCR reaction was performed in triplicates. The following PCR program was applied: initial denaturation: 95°C 10 min; 45 cycles: 95°C 15 s, 60°C 30 s, 72°C 30 s; final step: melting curve program. PCR primers were designed using Primer3 software [11]: (5′-3′): Tissue non-specific alkaline phosphatase, ALP (F: GGAAATCTGTGGGCATTGTG, R: CCCTGATGTTATGCATGAGC); Peroxisome proliferator-activated receptor gamma, PPARγ (F: TGCAGTGGGGATGTCTCATA, R: CAGCGGGAAGGACTTTATGT); Trancription factor SOX-9, SOX9 (F: GAGGAAGTCGGTGAAGAACG, R: AAGTCGATAGGGGGCTGTCT); Ribosomal protein L13, RPL13 (F: CCTGGAGGAGAAGAGGAAAGAGA, R: TTGAGGACCTCTGTGTATTTGTCAA). PCR primers were purchased from Life Technologies. Relative gene expression levels of lineage-specific markers in differentiated and undifferentiated cells were quantified by the following alogorithm: E^-Δct^. RPL13 served as reference gene.

### Flow cytometric cell cycle analysis

Cell harvests were resuspended in PBS^++^ at a concentration of 10^6^ cells/ml and fixed in ice cold (−20°C) 70% ethanol for at least 2 h at 4°C. Next, cells were washed in PBS^++^ and stained with PI solution (0.1% TritonX100, 10 μg/mL PI (Sigma-Aldrich), 100 μg/mL RNase A (Carl Roth) in PBS^++^) for 30 min at RT in the dark. Cellular DNA content was assessed through PI fluorescence, which was determined in a BD FACSCalibur flow cytometer. Fifteen thousand events were acquired in a pre-set “single cell” gate that excluded cell aggregates, and were analyzed using FlowJo software.

### CFU-F clonal assay

CB-MSC were subcultured, suspended in DMEM supplemented with 20% FCS (to ensure efficient attachment) and seeded at a density of 2 cells/cm^2^ into 6-well plates. Subsequent to visual inspection of seeding success, FCS was replaced by 10% human HF or control serum. After 14 days of treatment, cells were washed with PBS^++^, fixed with cold (4°C) 4% PFA for 30 min at RT, and were stained with 0.5% crystal violet solution (Carl Roth) for 30 min at RT on a rocking shaker. Afterwards excess stain was washed off and plates were air dried. Quantitative (number) and qualitative (size) CFU-F data was obtained from triplicate wells. All events seen in the plates (single cells, clusters comprised of 2, 4, 8, >10, >20, >50, >100, >200 or >500 cells) were counted.

### AnnexinV flow cytometric apoptosis detection

Cell harvests and supernatants were used to determine apoptosis by AnnexinV FITC Apoptosis Detection Kit I (BD Biosciences) according to the manufacturer’s instructions. Necrotic cells were discriminated by PI counterstaining. Fifteen thousand events were acquired in a BD FACSCalibur flow cytometer and analyzed using FlowJo software.

### Western blot

After 5 days of human serum treatment total protein from CB-MSC was isolated and quantified using Pro-Prep Protein Extraction solution (Intron Biotechnology, South Korea) and DC Protein Assay (Bio-Rad, Hercules, CA, USA) or BCA Protein Assay Kit (Pierce/Thermo Scientific, Rockford, IL, USA). Protein (15–20 μg) was separated by discontinuous SDS-PAGE and blotted to 0.45 μm nitrocellulose membranes (Bio-Rad, Carl Roth) by o/n wet tank or 40 min semidry transfer. Membranes were blocked for 1.5 h at RT in 5% skim milk prepared in TBS-T (10 mM TrisHcl pH 7.4, 100 mM NaCl, 0.1% Tween20 in ddH_2_O), washed with TBS-T and incubated with primary antibody o/n at 4°C, washed with TBS-T again and were stained with HRP- or IRDye-labeled secondary antibodies for 1 h at RT. Chemiluminescent protein detection was performed using the Amersham™ ECL Western Blotting Detection Reagents (GE HEALTHCARE, Little Chalfont, UK) and the FluorChem® HD2 Imaging System (Alpha Innotech, Santa Clara, CA, USA). Infrared protein detection was performed using the Odyssey Infrared Imaging System (LI-COR Biosciences, Lincoln, NE, USA). Primary mouse or rabbit antibodies were: Upstate/Millipore (Billerica, MA, USA): Bax; Abcam (Cambridge, UK): alpha Tubulin, p21, p16, p38, Caspase 3; Millipore: GAPDH, pp38; Cell Signaling (Danvers, MA, USA): CDK1, Cyclin E2, Cyclin D1, Cyclin B1, p27, JNK, pJNK, ERK1/2, pERK1/2, GAPDH; Santa Cruz Biotechnology (Santa Cruz, USA): CDK4, CDK2, pp53. Secondary goat antibodies were: anti-rabbit or anti-mouse IgG-HRP (Zymed/Life Technologies) and IRDye®680LT anti-mouse IgG or IRDye®800CW anti-rabbit IgG (LI-COR Biosciences). Band quantification was performed by ImageJ (NIH, Bethesda, MD, USA) as described elsewhere [12] (FCS treated cells served as standard).

### Statistics

Unless otherwise stated, medians (IQR) given were obtained from results of experiments performed with group sizes of at least four separate control or HF sera. Normal distribution provided, ANOVA with Bonferroni’s post-hoc test was done, otherwise the Kruskal-Wallis-Test or Mann–Whitney U-Test were applied to test for differences between groups. A general linear model was constructed to test the impact of several clinical variables on CB-MSC behavior within the HF patient cohort (see below). Statistical analyses were performed in SPSS 20 (IBM Corporation, Somers, NY, USA).

## Results

### HF serum cytokines

**Figure 1:**
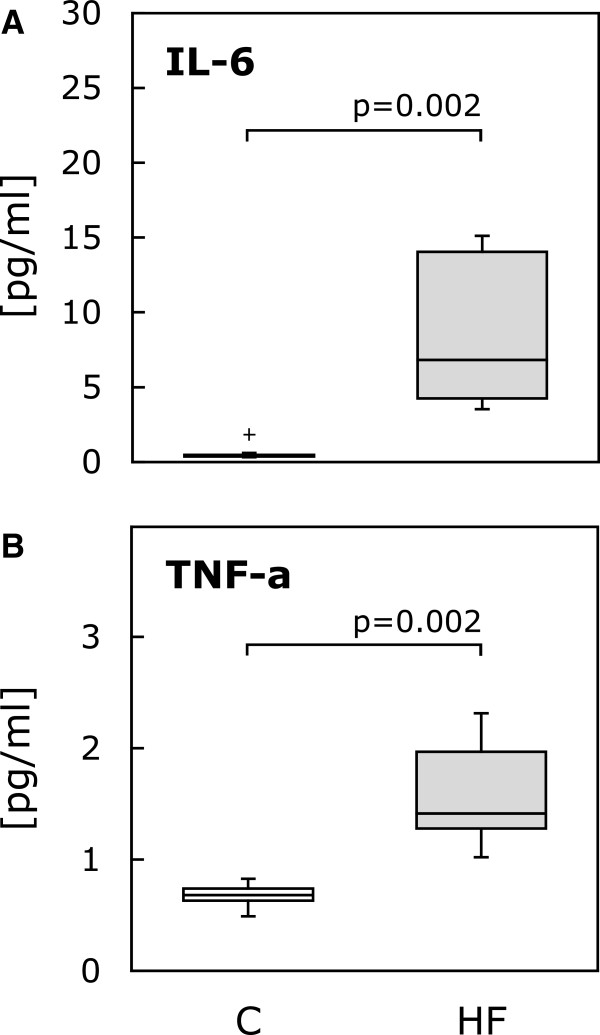
**HF serum biomarkers.** Serum cytokines in HF patients measured by high sensitivity ELISA. Compared to human control serum, both IL-6 **(A)** and TNF-α levels **(B)** were significantly higher in all HF sera. The boxplots indicate interquartile range (box), median (line), range (whiskers) and outliers (+/−).

Concentrations of IL-6 and TNF-α, common HF biomarkers, were determined by high sensitivity ELISA (Figure 1). Compared to human control serum, IL-6 and TNF-α concentrations were significantly higher in all HF sera (IL-6: control, 0.42 pg/ml (0.37-0.47 pg/ml) vs. HF, 6.83 pg/ml (4.26-14.04 pg/ml), p = 0.002; TNF-α: control, 0.68 pg/ml (0.63-0.74 pg/ml) vs. HF, 1.41 pg/ml (1.28-1.97 pg/ml), p = 0.002). However, we were not able to demonstrate a significant correlation between cytokine content and CB-MSC proliferation or apoptosis in individual sera (see below).

### HF serum treatment does not affect basic MSC characteristics

**Figure 2:**
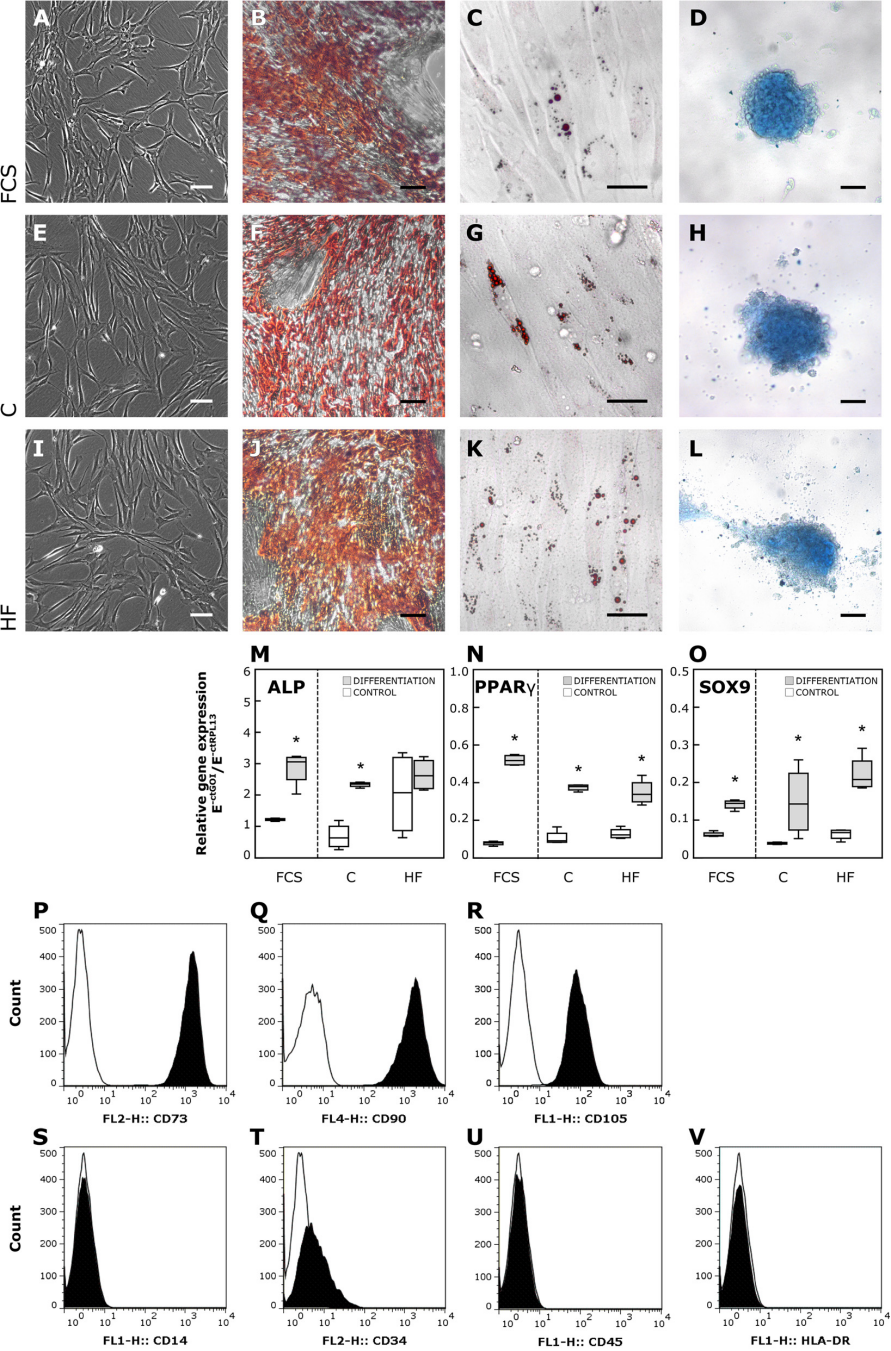
**Basic MSC properties in response to HF serum treatment. A**, **E**, **I**: Phase contrast imaging of CB-MSC morphology did not reveal obvious differences between FCS, the human control and the HF serum group. Scale bar = 100 μm. **B**-**D**, **F**-**H**, **J**-**L**: Tripotential mesodermal differentiation capacity: Osteogenesis (calcium deposits) was visualized by Alizarin Red S **(B**, **F**, **J)** (bar = 250 μm), adipogenesis (lipid vesicles) by Oil Red O **(C**, **G**, **K)** (bar = 50 μm) and chondrogenesis (proteoglycans) by Alcian Blue staining **(D**, **H**, **L)** (bar = 125 μm). **M**-**O**: Relative gene expression of lineage-specific markers in undifferentiated and differentiated cells: osteogenesis, ALP **(M)**, adipogenesis, PPARγ **(N)**, chondrogenesis, SOX9 **(O)**. The boxplots indicate interquartile range (box), median (line), range (whiskers) and outliers (+/−). * p < 0.05 vs. undifferentiated cells. GOI: gene of interest. **P**-**V**: Surface marker expression pattern of CB-MSC, as examined by flow cytometry, was not affected by HF serum treatment. Shown are histograms for CD73 **(P)**, CD90 **(Q)**, CD105 **(R)**, CD14 **(S)**, CD34 **(T)**, CD45 **(U)** and HLA-DR **(V)**, in which cell counts are plotted against fluorescence intensity. All histograms are representative for both human serum groups and the FCS control.

After 5 days of HF serum treatment, CB-MSC had retained their spindle-shaped morphology and plastic adherence (Figure 2A, E, I). Flow cytometric analysis of CB-MSC surface marker expression revealed neither qualitative nor significant quantitative differences between the three groups after 5 days of treatment (Figure 2P-V). In all cell populations analyzed, at least 98% of live cells expressed CD73, CD90 and CD105; less than 2% of live cells expressed CD14, CD34, CD45 and HLA-DR. Differentiation of CB-MSC towards the osteogenic, adipogenic and chondrogenic lineage was successfully performed for all cultures (human HF, control serum and FCS), as shown by the respective stainings (Figure 2B-D, F-H, J-L). Gene expression analysis of lineage-specific markers confirmed that differentiation induced the upregulation of ALP (osteogenesis), PPARγ (adipogenesis) and SOX9 (chondrogenesis) in all groups (relative gene expression in differentiated vs. undifferentiated cells, p = 0.03 for all groups and all markers). Interestingly, ALP expression in HF serum treated undifferentiated cells was elevated compared to undifferentiated cells treated with human control serum or FCS, and did not increase further during osteogenic differentiation (Figure 2M-O). Quantitatively, the osteogenic and chondrogenic differentiation potential did not differ between the human HF, control serum and the FCS group, as there was no relevant difference of lineage-specific marker expression in differentiated cells of all groups (ALP, p = 0.6; SOX9, p = 0.1). Solely, PPARγ expression in adipogenic differentiated HF serum treated cells was markedly lower compared to the FCS control (p = 0.02).

### HF alters clonogenicity and colony formation pattern of CB-MSC

**Figure 3:**
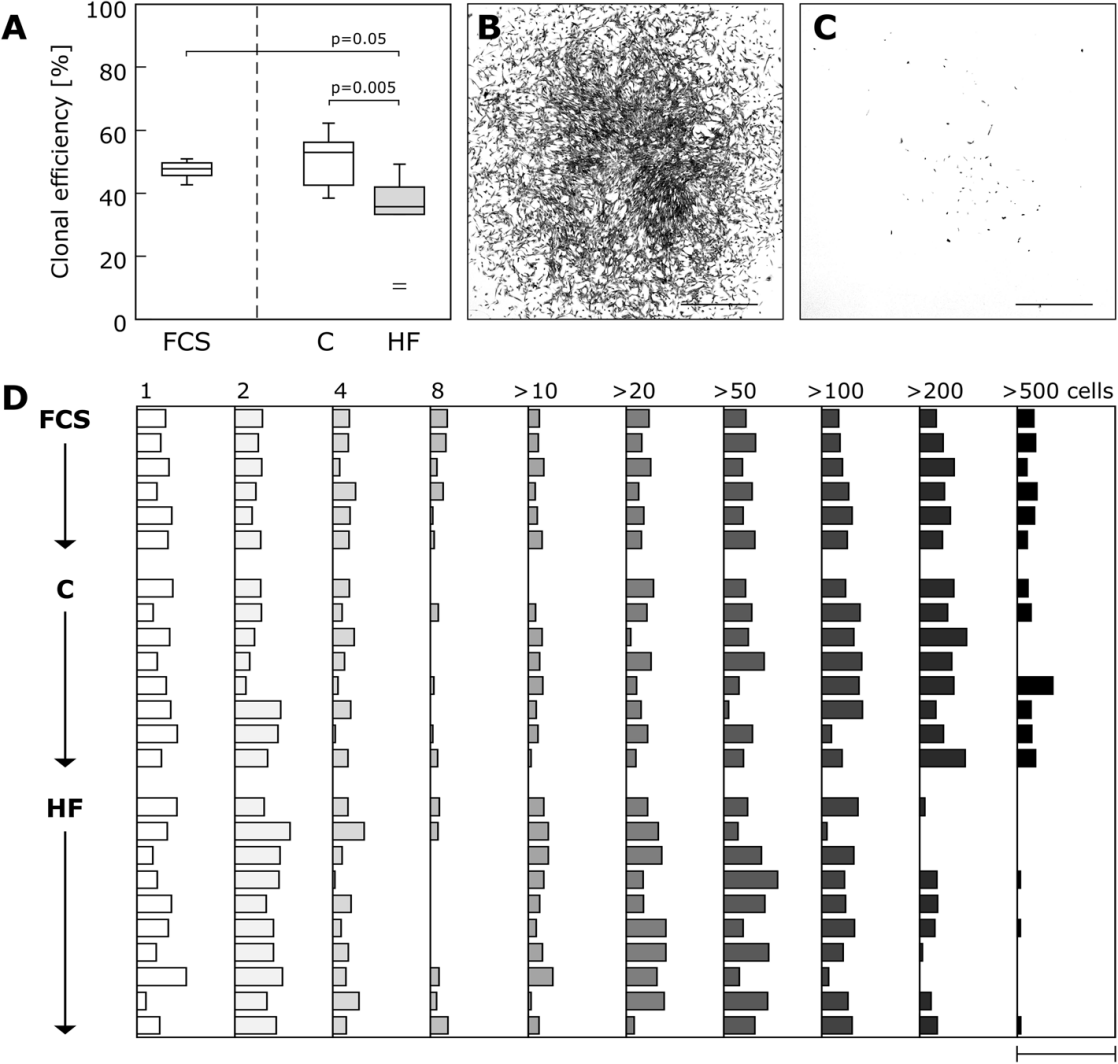
**Clonogenicity and colony pattern of CB-MSC in response to HF.** Clonal capacity was determined by CFU-F assay. **A**: Clonal efficiency in percent (number of colonies comprising more than 50 cells per number of plated cells) for each experimental group. CB-MSC clonal efficiency was significantly reduced in the HF group. The boxplot indicates interquartile range (box), median (line), range (whiskers) and outliers (+/−). **B**-**D**: Relative frequencies of cell cluster size categories in percent for single samples of all groups **(D)**. In the HF group there was an increase in the number of small (<50 cells) and a decrease in the number of large (>200, >500 cells) clusters. Bar = 50%. Representative bright field images of a typical large and a small cluster **(B**, **C)**. Scale bars = 1 mm.

CB-MSC clonogenicity was examined by colony forming unit-fibroblast (CFU-F) assay. The quantification of clonal efficiency (number of colonies comprising more than 50 cells per number of initially plated cells) showed a significantly lower percentage of clonogenic cells in the HF group (human control, 53.0% (42.6-56.2%) vs. HF, 35.7% (33.3-42.0%), p = 0.005; FCS, 47.8% (45.7-49.7%) vs. HF, p = 0.05; Figure 3A). Moreover, the quantitative analysis of colony size revealed a difference in the pattern of cluster size between the treatment groups. Notably, in the presence of HF serum CB-MSC formed more small-sized clusters (composed of <50 cells) but fewer large-sized clusters (composed of >200 cells) than in human control serum or FCS (Figure 3B-D).

### Impaired proliferation of CB-MSC upon HF serum treatment

**Figure 4:**
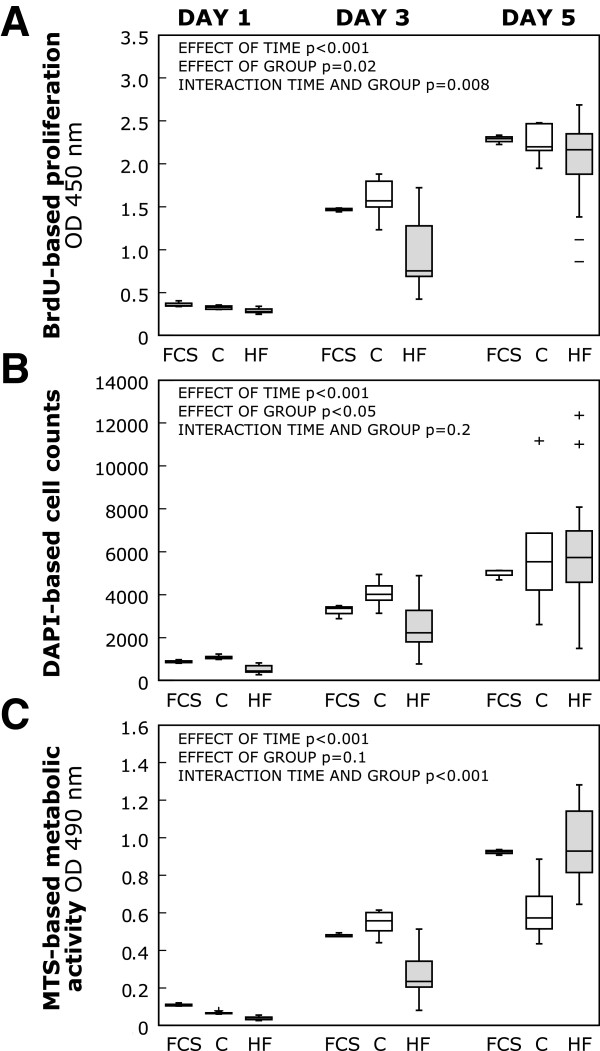
**CB-MSC proliferation in response to HF serum.** To assess CB-MSC kinetics of proliferation and activity in response to individual sera, three independent assays were performed with P4 cells after 1, 3 and 5 days of treatment. **A**: DNA synthesis levels were obtained from BrdU assay. Graphs show optical densities recorded at 450 nm (OD 450 nm) for all treatment groups. **B**: DAPI-based cell counting approach for direct quantification of CB-MSC number. **C**: Metabolic activity determined by MTS assay, given as optical density at 490 nm. In all assays, CB-MSC proliferation was impaired after 1 and 3 days, but not after 5 days of treatment with HF serum as compared with human control serum treatment. The effect of time and group and their interaction were determined by ANOVA for repeated measurements. The boxplots indicate interquartile range (box), median (line), range (whiskers) and outliers (+/−).

Here, we sought to elucidate the kinetics of cellular proliferation and metabolic activity in greater detail. Irrespective of the type of serum, CB-MSC grew steadily, as evident from the readouts of three independent proliferation assays (Figure 4), but we observed marked differences in growth rate between the groups. Overall, CB-MSC proliferation was significantly suppressed in the HF group during the first 3 days of treatment and accelerated during the final phase of observation. In human control serum and FCS, growth rate was high in the beginning and consequently reached saturation earlier. In the BrdU cell proliferation assay, CB-MSC DNA synthesis levels were significantly lower after 1 and 3 days of exposure to HF serum as compared to human control serum (Figure 4A). After 5 days, however, the proliferation status of HF serum treated CB-MSC approached that of control serum treated CB-MSC at 5 days. The DAPI-based direct cell counting method confirmed this observation (Figure 4B). To further assess the metabolic activity of CB-MSC, a MTS assay was performed. Corresponding to proliferation behaviour seen with BrdU and DAPI counting, metabolic activity was significantly reduced within the first 3 days of treatment. By day 5, however, HF serum treated CB-MSC reached metabolic activity levels that were significantly higher than those reached with human control serum, and comparable to those with FCS (Figure 4C).

### Cell cycle progression in CB-MSC is inhibited in the presence of HF serum

**Figure 5:**
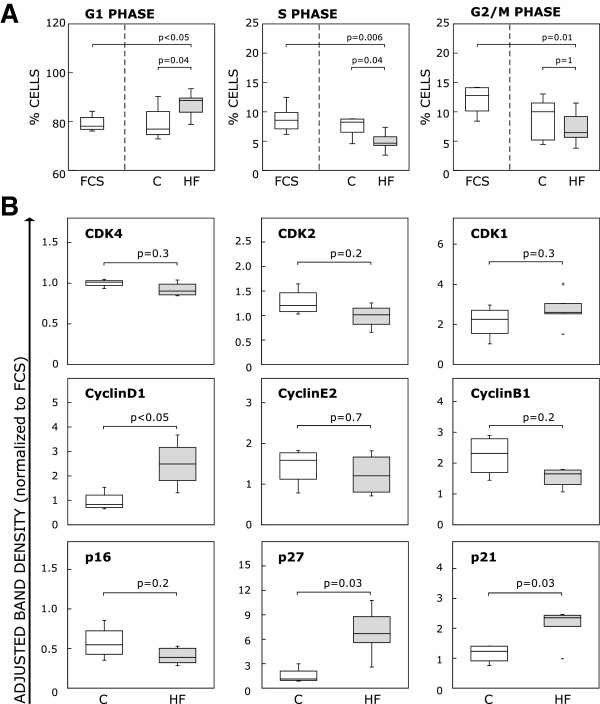
**Cell cycle state of CB-MSC upon HF and control serum treatment. A**: Cell cycle status of individual cells was determined after 5 days of HF serum treatment by flow cytometric analysis of DNA content of cells stained with PI. The graphs depict the percentage of cells present in each cell cycle phase. Compared to the human control serum and the FCS group, the number of actively cycling cells in the HF group was significantly lower. **B**: After 5 days of treatment with either human control, HF serum or FCS, protein expression levels of cyclin dependent kinases (CDK), their positive (cyclins) and negative regulators (CDK inhibitors/CDKI of the INK4A and CIP/KIP family) involved in G1 progression (column 1), G1-S transition (column 2) and M phase entry (column 3) were determined by Western blot. Boxplots display adjusted band densities normalized to FCS for both human treatment groups. HF serum treatment blocks cell cycle progression of CB-MSC through upregulation of p21 and p27. The boxplots indicate interquartile range (box), median (line), range (whiskers) and outliers (+/−).

After 5 days of cultivation of CB-MSC in human serum, we determined the distribution of single cells within the three cell cycle phases (G0/G1, S, G2/M) by flow cytometric quantification of DNA content in cells stained with PI (Figure 5A). As expected, the majority (>70%) of cells were in the G0/G1 phase and the remaining cells were almost equally distributed between the S and G2/M phases. However, we found a difference between human HF and control serum or FCS regarding the percentage of cells in the G0/G1 and S phase. In the HF group there were significantly more cells in G0/G1 (human control, 76.9% (74.7-84.1%) vs. HF 88.6% (83.9-89.6%), p = 0.04; FCS, 78.1% (76.8-81.7%) vs. HF, p < 0.05) and fewer cells in the S phase (human control, 8.2% (6.5-8.8%) vs. HF, 4.6% (4.3-5.7%), p = 0.04; FCS, 8.6% (7.1-9.9%) vs. HF, p = 0.006). Interestingly, the percentage of cells in G2/M phase did only differ between the HF and the FCS group (human control, 10.0% (5.2-11.5%) vs. HF, 6.4% (5.6-9.2%), p = 1; FCS 12.8% (10.1-14.1%) vs. HF, p = 0.01).

### Insights into the molecular cell cycle machinery of HF serum treated CB-MSC

By immunoblotting, we monitored the expression of stage specific proteins that drive cell cycle progression and act in its regulation. We studied the expression of cyclin dependent kinases (CDK), their activating cyclins and their inhibitors (CDKI) contributing to G1 phase progression (CDK4, Cyclin D1, CDKI p16^INK4A^), G1-S phase transition (CDK2, Cyclin E2, CDKI p21^CIP1^ and p27^KIP1^) and M phase entry (CDK1, Cyclin B1, CDKI p21^CIP1^). The data are summarized in Figure 5B. While Cyclin D1 expression was elevated and p16 unchanged in response to HF serum (adjusted band densities: Cyclin D1: human control, 0.83 (0.71-1.2) vs. HF, 2.49 (1.81-3.17), p < 0.05; p16: human control, 0.55 (0.43-0.72) vs. HF, 0.39 (0.32-0.5), p = 0.2), completion of G1-S phase transition as well as the G2 and M phase of the cell cycle were clearly inhibited as evidenced by upregulation of p21 and p27, and a trend toward downregulation of Cyclin B1 (p21: human control, 1.23 (0.91-1.4) vs. HF, 2.35 (2.06-2.44), p = 0.03; p27: human control, 1.12 (0.94-2.09) vs. HF, 6.86 (5.59-8.77), p = 0.03; Cyclin B1: human control, 2.32 (1.70-2.79) vs. HF, 1.65 (1.31-1.78), p = 0.2). This pattern indicates that, in response to HF serum, most CB-MSC rest in the G1 phase of the cell cycle, and are not able to progress despite a certain pro-mitotic stimulation (Cyclin D1 upregulation).

### HF serum treatment triggers apoptosis in CB-MSC

**Figure 6:**
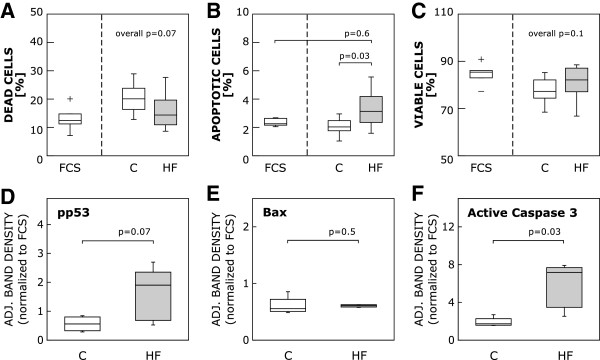
**HF serum provokes apoptosis in CB-MSC. A**-**C**: Plasma membrane alterations (translocation of phosphatidyl serine) were assessed by flow cytometric analysis of cells subjected to a combined staining with AnnexinV (AV) and Propidium Iodide (PI). Boxplots show the percentages of dead (PI+) **(A)**, apoptotic (AV+, PI-) **(B)** and viable cells (AV-, PI-) **(C)**. There were significantly more apoptotic cells in the HF group. **D**-**F**: Western blots were performed to determine intracellular amounts of pro-apoptotic proteins, such as phospho-p53 **(D)**, Bax **(E)** and active Caspase 3 **(F)**. Boxplots illustrate quantified and adjusted band densities normalized to FCS for the human serum groups. Compared to the human control serum group, the HF group exhibited markedly higher levels of Caspase 3, whereas Bax expression did not differ. The boxplots indicate interquartile range (box), median (line), range (whiskers) and outliers (+/−).

We also sought to investigate the role of apoptotic events in HF serum treated CB-MSC cultures and studied plasma membrane alterations and the expression of proteins involved in the initiation and execution of CB-MSC death (Figure 6). Flow cytometric analysis of AnnexinV stained cells (AnnexinV + and PI-) revealed a significantly higher number of cells that had translocated phosphatidyl serine to the outer surface of their plasma membranes in the HF group (human control, 2.03% (1.77-2.48%) vs. HF, 3.13% (2.35-4.18%), p = 0.03; FCS, 2.26% (2.16-2.64%) vs. HF, p = 0.6). By PI staining, the proportion of necrotic cells (PI+) was not significantly different between human control, HF serum and FCS treated cells, neither was the percentage of viable (AnnexinV- and PI-) cells (necrotic cells: overall p = 0.07; viable cells: overall p = 0.1). Western blot analysis of pro-apoptotic proteins showed markedly increased protein levels of cleaved Caspase 3 in the HF treatment group after 5 days of exposure (adjusted band densities: human control, 1.74 (1.58-2.28) vs. HF, 7.16 (3.48-7.69), p = 0.03). While the intergroup difference in phosphorylated p53 expression indicated a trend towards increased levels (p = 0.07), but lacked definitive statistical significance, Bax protein expression did not differ between the groups (adjusted band densities: phospho-p53: human control, 0.56 (0.32-0.8) vs. HF, 1.9 (0.68-2.35), p = 0.07; Bax: human control, 0.56 (0.51-0.72) vs. HF, 0.61 (0.58-0.63), p = 0.5).

### ERK1/2 and JNK transduce the HF serum cytokine stimulus into CB-MSC response

**Figure 7:**
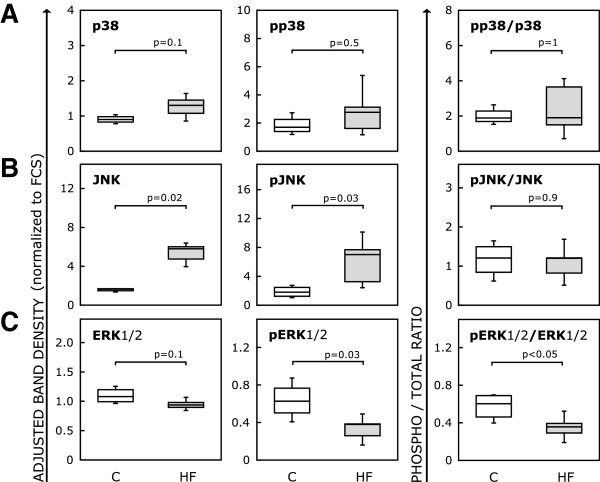
**Signal transduction in CB-MSC in response to HF and control serum.** Immunoblot analyses were performed for p38 **(A)**, JNK **(B)** and ERK1/2 **(C)** after 5 days of CB-MSC culture in medium supplemented with either human control, HF serum or FCS. Boxplots show expression levels of total or phosphorylated proteins as adjusted band densities normalized to FCS and the calculated phosphorylation status plotted as ratio of phosphorylated-to-total protein for both human serum groups. In response to HF serum, JNK seemed to be transcriptionally upregulated, whereas ERK1/2 phosphorylation was clearly reduced. The boxplots indicate interquartile range (box), median (line), range (whiskers) and outliers (+/−).

Serum from HF patients contains a variety of factors that may bind to plasma membrane receptors and initiated intracellular signaling cascades leading to a proliferation block and apoptosis. Typically, such stress signals are relayed by mitogen activated protein (MAP) kinases, which activate a variety of transcription factors. We therefore studied the expression and phosphorylation of the three downstream effector MAP kinases, ERK1/2, JNK and p38. As seen in Figure 7, p38 protein expression remained unchanged in response to HF serum, but JNK total protein expression (adjusted band densities: human control, 1.65 (1.49-1.69) vs. HF, 5.80 (4.75-6.01), p = 0.02) and phosphorylated JNK (human control, 1.81 (1.25-2.47) vs. HF, 7.01 (3.26-7.68), p = 0.03) were significantly elevated, while the phosphorylated-to-total ratio of ERK1/2 was reduced (human control, 0.6 (0.46-0.69) vs. HF, 0.36 (0.29-0.39), p < 0.05). Usually JNK is activated by phosphorylation only, resulting in a higher ratio of phosphorylated-to-total JNK, while here there was clearly also transcriptional upregulation of JNK expression, reflecting the magnitude of stress induced by HF serum and the extended period of time the cells were subjected to it.

### Heart failure subgroup analysis

**Figure 8:**
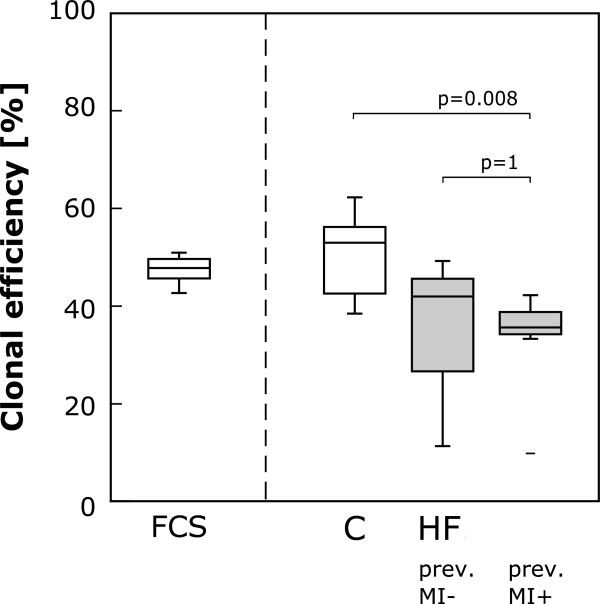
**Impact of clinical variables on CB-MSC behavior.** Clonal efficiency stratified according to the presence of myocardial infarction (MI) in the patient’s history. Here, only serum from patients with previous MI has signficantly reduced clonality. Please note the limitations of this subgroup analysis that are discussed in the text. The boxplot indicates interquartile range (box), median (line), range (whiskers) and outliers (+/−).

We also studied the impact of clinical variables within the HF patient cohort on the clonogenicity and proliferative capacity of CB-MSC. Specifically, patient age, gender, ischemic vs. non-ischemic heart disease, LVEF, IL-6 and TNF-α serum levels and the need for mechanical circulatory support were tested as continuous or categorical variables, respectively. In the multivariate analysis, none of the clinical variables had significant impact on CB-MSC behavior. However, when the data were stratified according to the presence of previous myocardial infarction, we found that, compared with serum from healthy control subjects, clonal efficiency was only reduced in serum from patients with previous myocardial infarction (MI) (Figure 8).

## Discussion

Since the regenerative capacity of HF-patient derived stem cells is limited through age and disease [13,14], the use of neonatal cell products for cardiac cell therapy may represent a better alternative. It is, however, unknown whether the performance of these juvenile cells would be affected by the various stresses imposed by the diseased organism. We therefore investigated the behavior of CB-MSC in response to serum from patients with severe HF. CB-MSC maintained their principal MSC characteristics including surface marker phenotype, osteogenic, chondrogenic and limited adipogenic differentiation capacity. This robustness of the minimal MSC criteria has also been shown for adult hBM-MSC in autologous and allogeneic human serum from healthy individuals [15], as well as in the presence of autologous human serum from patients with heart disease [16]. Only under more forceful manipulation of MSC *in vitro*, such as exposure of MSC to local anesthetics or replicative senescence in high passages do MSC display detectable changes in morphology and differentiation capacity [17,18]. While the minimal MSC properties withstand most external factors, clonality and proliferative capacity are more susceptible. In our experiments, clonal efficiency was reduced in the presence of HF serum, and the colonies that did develop were predominantly small. Thus, within a CB-MSC population some cells are inhibited to divide at all, whereas others are significantly slowed down in doing so. Similar phenomena have been described for cells of endothelial and hematopoietic lineage, where therapeutic interventions in patients partially reversed the inhibitory serum effects [6,19]. Interestingly, the colony-forming ability of endogenous hBM-MSC seems to be activated in patients with cardiovascular risk factors, but the majority of the patients in that study had coronary artery disease but not HF [16].

Similar to the results obtained from the CFU-F assay, CB-MSC proliferation as measured by BrdU incorporation, direct cell counting and MTS test was initially inhibited by HF serum. This was associated with strong upregulation of cell cycle inhibitors p21 and p27 that block progression to the S, G2 and M phase. Accordingly, the majority of CB-MSC rested in the G1 phase. Our finding of concomitant Cyclin D1 upregulation indicates attempted or beginning mitosis and may reflect the reversal of the proliferation block at day 5. Overall, the HF induced CB-MSC depression is in line with observations made by Yamahara *et al.*[5], who used adult hBM-MSC and identified Angiostatin as one mediator of HF induced MSC depression. In our experiments, however, MSC proliferation kinetics caught up with and metabolic activity even exceeded those observed in human control serum around day 5. The most straightforward explanation for this phenomenon is that MSC adapt to the presence of HF serum factors and compensate for the initial proliferation inhibition. Possible mechanisms include downregulation of surface receptors involved, as in agonist-induced desensitization of, for instance, adrenergic receptors. In addition to attenuation of inhibitory signaling, activation of stimulatory intracellular mechanisms may also play a role. It may be argued that methodological factors such as degradation of serum factors in the culture medium also played a role, but the medium was completely replaced every 48 hours.

HF serum treatment also triggered MSC apoptosis, as evidenced by increased phosphatidyl serine exposure and Caspase 3 activation. Such a pro-apoptotic effect of HF serum has also been noted by Mammi *et al.*, who used endothelial cells from healthy donors as a model [20]. Expression of Bax (Bcl-2-associated X protein), which is involved in mitochondrial membrane permeabilization, was not upregulated in our model. One explanation for this contradictory finding is that upon activation Bax changes its conformation, translocated to the outer mitochondrial membrane, and induced cytochrome C release, so that transcriptional activation is not required to induce or accelerate apoptotic cell death (reviewed in [21]).

Regarding the intracellular signaling pathways, it is well established that MAP kinases relay stress signals upon extracellular stimulation with, for instance, growth factors and cytokines, and among the targets of MAP kinases are regulators of proliferation and apoptosis [22]. The downstream effector MAP kinases are ERK1/2, JNK and p38, all of which directly activate nuclear transcription factors. We found a substantial downregulation of ERK1/2 phosphorylation, possibly due to lower levels of growth factors in HF sera, which may account for impaired proliferation in CB-MSC. We also found that total expression of JNK as well as the amount of phosphorylated JNK were significantly elevated, so that the ratio of phosphorylated to total JNK remained constant despite clear activation of the pathway. This transcriptional regulation of JNK is unusual and underscores the profound effect of HF serum on CB-MSC. Activation of p38, however, does not seem to play a major role. This finding may surprise because p38 is typically strongly induced by inflammatory cytokines, while JNK mediates cellular response to environmental stresses (oxidative stress and DNA damage for instance). MAP kinase pathways leading to JNK are typically activated by FAS death receptors, but also by inflammatory cytokine receptors including TNF-α and TGF-β. A more detailed analysis of HF induced signaling events in somatic stem cells may be necessary in the future.

It was beyond the scope of this study to identify the components of HF serum that are responsible for the temporary MSC depression. As mentioned above, Yamahara *et al*. described a prominent role of Angiostatin in the inhibition of hBM-MSC by serum from patients with HF [5]. However, it may be safe to assume the effect of HF serum on CB-MSC biology is the result of concerted cytokine actions within a tremendous protein pool. In addition, drugs used for palliative treatment of HF, neurohormones and reactive oxygen species may also play a role [4].

### Limitations of the study

The *in vitro* setting we chose for our experiments is clearly a very artificial environment that is far from mimicking the situation *in vivo*, especially in man. Further studies in long-term *in vitro* settings as well as *in vivo* are needed to solidify the evidence. Working with pooled patient sera may have provided more robust data in some experiments, but we chose to test the sera separately in order to be able to test for patient-specific confounding factors. Several groups that have pioneered the isolation and characterization of CB-MSC used higher FCS concentrations in their protocols, so that one may suspect that our cells were suffering from low serum concentrations throughout [23]. We chose to conduct our experiments with 10% serum because, first, the primary CB-MSC cultivation and expansion (K.B.) was done this way, and, second, we had only a limited volume of patient serum available for our experiments. It is not possible, either, to infer from our data what the most important molecular stimuli of the depressive HF effects are. In addition to having heart failure, our patients received a multitude of different pharmaceuticals, of which each may interfere with CB-MSC function. Again, further studies would be needed to dissect these effects. In the univariate analysis we found that serum from patients with previous myocardial infarction may inhibit CB-MSC more than that of HF patients without MI (Figure 8). However, these data must be interpreted with caution, because cohort size is very small after stratification. Finally, we worked with a multitude of sera but all our CB-MSC were from the same batch. We chose this approach to limit possible confounding factors with CB-MSC and feel that this is justified because neonatal cells are very uniform and have not been subjected to age, disease and lifestyle-related changes. However, confirmation of our data in other CB-MSC batches would clearly be necessary if immediately relevant conclusions were to be drawn.

## Conclusion

We found that serum from patients with advanced HF temporarily inhibits the proliferative performance of “healthy” neonatal cord blood-derived MSC. This proliferation block seems to be overcome after 5 days in our experimental setting, but reduced number of colony-forming units and upregulation of several molecular cell cycle inhibitors remain detectable beyond that point. These data confirm the inhibitory impact of HF on stem cell populations, and demonstrate that even allogeneic neonatal cells are affected by this phenomenon. Although our experiments were performed in an artificial *in vitro* setting, this kind of study gives valuable insight into the behavior of exogenous therapeutic cell products in response to the diseased recipient organism. Ultimately, such studies may lead to the development of personalized, patient-specific cell therapies that are adapted to the individual patient’s response.

## Competing interests

All authors state that they have no conflicts of interests.

## Authors’ contributions

Conception of the study: CS, KK. Design of the study: KK, AK, KSK, YHC, CS. Provision of CB-MSC: KB. Provision of PCR-Primers: AO. Acquisition of data: KK, RR. Supervision of experiments: AB. Analysis and interpretation of data: KK, AK, AO, KSK, KB, YHC, CS. Drafting the manuscript: KK, CS. Revision of the manuscript: KK, KB, YHC, CS. All authors read and approved the final manuscript.
